# Association study of human leukocyte antigen variants and idiopathic pulmonary fibrosis

**DOI:** 10.1183/23120541.00553-2023

**Published:** 2024-02-19

**Authors:** Beatriz Guillen-Guio, Megan L. Paynton, Richard J. Allen, Daniel P.W. Chin, Lauren J. Donoghue, Amy Stockwell, Olivia C. Leavy, Tamara Hernandez-Beeftink, Carl Reynolds, Paul Cullinan, Fernando Martinez, Helen L. Booth, William A. Fahy, Ian P. Hall, Simon P. Hart, Mike R. Hill, Nik Hirani, Richard B. Hubbard, Robin J. McAnulty, Ann B. Millar, Vidya Navaratnam, Eunice Oballa, Helen Parfrey, Gauri Saini, Ian Sayers, Martin D. Tobin, Moira K.B. Whyte, Ayodeji Adegunsoye, Naftali Kaminski, Shwu-Fan Ma, Mary E. Strek, Yingze Zhang, Tasha E. Fingerlin, Maria Molina-Molina, Margaret Neighbors, X. Rebecca Sheng, Justin M. Oldham, Toby M. Maher, Philip L. Molyneaux, Carlos Flores, Imre Noth, David A. Schwartz, Brian L. Yaspan, R. Gisli Jenkins, Louise V. Wain, Edward J. Hollox

**Affiliations:** 1 Department of Population Health Sciences, University of Leicester, Leicester, UK; 2 NIHR Leicester Biomedical Research Centre, Leicester, UK; 3 Genentech, San Francisco, CA, USA; 4 National Heart & Lung Institute, Imperial College London, London, UK; 5 Department of Medicine, Weill Cornell Medicine, New York, NY, USA; 6 University College Hospital, University College London, London, UK; 7 GlaxoSmithKline, London, UK; 8 School of Medicine, University of Nottingham, Nottingham, UK; 9 National Institute for Health Research, Nottingham Biomedical Research Centre, Nottingham, UK; 10 Hull York Medical School, University of Hull, Hull, UK; 11 MRC Population Health Unit, University of Oxford, Oxford, UK; 12 Centre for Inflammation Research, University of Edinburgh, Edinburgh, UK; 13 Division of Medicine, University College London, London, UK; 14 Bristol Medical School, University of Bristol, Bristol, UK; 15 Division of Epidemiology and Public Health, University of Nottingham, Nottingham, UK; 16 National Institute for Health Research, Nottingham Biomedical Research Centre, Nottingham University Hospitals NHS Trust, Nottingham, UK; 17 Queensland Lung Transplant Service, The Prince Charles Hospital, Brisbane, QLD, Australia; 18 Royal Papworth Hospital NHS Foundation Trust, Cambridge, UK; 19 Centre for Respiratory Research, NIHR Nottingham Biomedical Research Centre, School of Medicine, Biodiscovery Institute, University of Nottingham, Nottingham, UK; 20 Department of Medicine, University of Chicago, Chicago, IL, USA; 21 Pulmonary, Critical Care and Sleep Medicine, Yale School of Medicine, New Haven, CT, USA; 22 Department of Medicine, University of Virginia, Charlottesville, VA, USA; 23 Division of Pulmonary, Allergy, Critical Care, and Sleep Medicine, University of Pittsburgh, Pittsburgh, PA, USA; 24 Department of Immunology and Genomic Medicine, National Jewish Health, Denver, CO, USA; 25 Servei de Pneumologia, Laboratori de Pneumologia Experimental, Instituto de Investigación Biomédica de Bellvitge (IDIBELL), Barcelona, Spain; 26 Campus de Bellvitge, Universitat de Barcelona, Barcelona, Spain; 27 Centro de Investigación Biomédica en Red de Enfermedades Respiratorias (CIBERES), Instituto de Salud Carlos III, Madrid, Spain; 28 Division of Pulmonary and Critical Care Medicine, University of Michigan, Ann Arbor, MI, USA; 29 National Heart and Lung Institute, Imperial College London, London, UK; 30 Division of Pulmonary and Critical Care Medicine, University of Southern California, Los Angeles, USA; 31 Royal Brompton and Harefield Hospitals, Guy's and St Thomas’ NHS Foundation Trust, London, UK; 32 Research Unit, Hospital Universitario Nuestra Señora de Candelaria, Santa Cruz de Tenerife, Spain; 33 Genomics Division, Instituto Tecnologico y de Energias Renovables, Santa Cruz de Tenerife, Spain; 34 Facultad de Ciencias de la Salud, Universidad Fernando Pessoa Canarias, Las Palmas de Gran Canaria, Spain; 35 Department of Medicine, University of Colorado, Anscuhtz, CO, USA; 36 Department of Genetics and Genome Biology, University of Leicester, Leicester, UK; 37 Joint first authors; 38 Joint senior authors

## Abstract

**Introduction:**

Idiopathic pulmonary fibrosis (IPF) is a chronic interstitial pneumonia marked by progressive lung fibrosis and a poor prognosis. Recent studies have highlighted the potential role of infection in the pathogenesis of IPF, and a prior association of the *HLA-DQB1* gene with idiopathic fibrotic interstitial pneumonia (including IPF) has been reported. Owing to the important role that the human leukocyte antigen (HLA) region plays in the immune response, here we evaluated if HLA genetic variation was associated specifically with IPF risk.

**Methods:**

We performed a meta-analysis of associations of the HLA region with IPF risk in individuals of European ancestry from seven independent case–control studies of IPF (comprising 5159 cases and 27 459 controls, including a prior study of fibrotic interstitial pneumonia). Single nucleotide polymorphisms, classical HLA alleles and amino acids were analysed and signals meeting a region-wide association threshold of p<4.5×10^−4^ and a posterior probability of replication >90% were considered significant. We sought to replicate the previously reported *HLA-DQB1* association in the subset of studies independent of the original report.

**Results:**

The meta-analysis of all seven studies identified four significant independent single nucleotide polymorphisms associated with IPF risk. However, none met the posterior probability for replication criterion. The *HLA-DQB1* association was not replicated in the independent IPF studies.

**Conclusion:**

Variation in the HLA region was not consistently associated with risk in studies of IPF. However, this does not preclude the possibility that other genomic regions linked to the immune response may be involved in the aetiology of IPF.

## Shareable abstract

**Variation in the HLA region is not consistently associated with risk in studies of IPF. However, this does not preclude the possibility that other genomic regions linked to the immune response may be involved in the aetiology of IPF.** https://bit.ly/477T8XN

## Introduction

Idiopathic pulmonary fibrosis (IPF) is a lung disease characterised by progressive scarring of the alveoli leading to impaired gas exchange. The median survival after IPF diagnosis is 2–5 years and there are limited drug treatments for patients [1]. Several studies have reported both environmental and genetic risk factors related to IPF [2]. However, the cause of IPF development remains unclear.

Genetic variation in the human leukocyte antigen (HLA) region, also known as the major histocompatibility complex (MHC) region, has been reported to be associated with inflammatory and respiratory diseases [3, 4]. This includes fibrotic idiopathic interstitial pneumonia (fIIP), where HLA-DQB1*06:02 has been associated with increased risk of disease [5]. Additionally, HLA-DRB1*15:01 has been found to be more prevalent among IPF patients than controls [6]. The mechanism behind these associations is unclear. However, it has been previously suggested that respiratory infections, including COVID-19, could trigger the development and progression of interstitial lung diseases, including IPF [7–9]. Indeed, antiviral drugs against herpesviruses have been proposed to attenuate disease progression in IPF patients [10]. Given the important role of the MHC receptors encoded by HLA genes in presenting viral antigens to the host immune system, genetic variation at the HLA region could influence the response to these infections and therefore IPF pathophysiology.

Recent studies on large biobanks have emphasised the pleiotropy of the HLA region [11]. This region is highly polymorphic and gene-dense, making the interpretation of single nucleotide polymorphism (SNP) associations difficult, and its study requires specific imputation techniques [12]. The latest SNP imputation panels enable improved imputation accuracy within the HLA region. Additionally, specific HLA imputation panels also allow the imputation of classical HLA alleles and amino acid alleles across the HLA region, which are biologically informative [13].

Given the potential role of infection response in IPF physiopathology, and the suggested association of HLA-DQB1*06:02 and HLA-DRB1*15:01 with disease, here we present the largest analysis of the HLA region in IPF patients, comprising seven independent case–control cohorts for IPF, with a total of over 5000 cases and 27 000 controls. We used an HLA-specific imputation approach to impute SNP variation, classical HLA alleles and amino acid alleles (hereafter referred to as variants) with the aim of identifying novel IPF risk loci within the HLA region that may influence the response to infections and therefore IPF pathophysiology.

## Methods

### Sample, genotyping and quality controls

We analysed genomic data from seven previously described independent case–control studies, named here as CleanUP-UCD (Study of Clinical Efficacy of Antimicrobial Therapy Strategy Using Pragmatic Design - University of California Davis) [14, 15], Colorado [16], Genentech [17, 18], IPF-JES (Job Exposures Study) [19], UK [20], US [21] and UUS (USA, UK and Spain) [22]. CleanUP-UCD, IPF-JES, UK, US and UUS studies included patients diagnosed with IPF as cases and population controls. The Colorado study used cases of fIIP (including IPF cases among other conditions) and population controls and was the study that previously reported the fIIP association for HLA-DQB1*06:02 [5]. The Genentech study included patients with IPF and controls from non-IPF clinical trials of age-related macular degeneration, diabetic macular oedema, multiple sclerosis, asthma and inflammatory bowel disease. In all seven studies, cases were diagnosed according to the American Thoracic Society and European Respiratory Society guidelines [23–27]. The studies were performed in accordance with The Code of Ethics of the World Medical Association (Declaration of Helsinki) and approved by the appropriate institutional review or research ethics committee. Further details about the seven studies can be found in the supplementary methods and table S1.

In the CleanUP-UCD, Colorado, IPF-JES, UK, US and UUS studies, individuals were genotyped using SNP arrays (Affymetrix and Illumina Inc.). Genotyping and quality control procedures have been previously detailed [20, 22, 28]. In summary, for these six studies, individuals were filtered based on low call rate estimates, sex mismatches, excess heterozygosity, relatedness and non-European ancestry (based on principal components (PCs) clustering with European individuals from The 1000 Genomes Project, www.internationalgenome.org). Individuals overlapping between studies were removed. Genotypes from individuals in the Genentech study were obtained from whole-genome sequencing using the HiSeq X Ten platform (Illumina Inc.) to an average read depth of 30×. Related individuals and those with genotype missingness >10% or excess heterozygosity were excluded. Ancestry was determined using ADMIXTURE v1.23 [29], and individuals with a European genetic-ancestry score >0.7 were included in the analyses.

### Variant imputation in the HLA region and association tests

For each study, classical HLA alleles, amino acid alleles and SNPs located within chr6: 28510120–33480577 (GRCh38) were imputed to capture genetic variation in the HLA region (supplementary table S1). Supplementary table S2 summarises the sequence of statistical analyses conducted in the study.

For the CleanUP-UCD, Colorado, IPF-JES, UK, US and UUS studies, phasing and SNP imputation to the TOPMed reference panel was performed using the TOPMed Imputation Server [30, 31]. Classical HLA alleles from class I genes (*HLA-A*, *HLA-B*, *HLA-C*) and class II genes (*HLA-DPA*, *HLA-DPB*, *HLA-DQA*, *HLA-DQB*, *HLA-DRB*), amino acid alleles and additional SNPs were imputed to the Type 1 Diabetes Genomics Consortium (T1DGC) panel [32] using IMPUTE2 (v2.3.2) (https://mathgen.stats.ox.ac.uk/impute/impute_v2.html) after chromosome phasing with SHAPE-IT v2.837 [33]. After SNP imputation using both TOPMed and T1DGC panels, duplicated SNPs were removed, prioritising those imputed with TOPMed. For the Genentech study, variants with a minor allele frequency (MAF) <1% and SNPs absent from the TOPMed reference panel were removed. Classical HLA alleles and amino acid alleles were imputed using HLA-HD [34], and data was analysed using MiDAS [35].

Logistic regression analyses were performed in each study separately assuming an additive model. For the CleanUP-UCD, Colorado, IPF-JES, UK, US and UUS studies, models were adjusted for the 10 leading PCs to correct for population stratification. For the Genentech study, sex, age and five genetic-ancestry PCs were included as covariates and reverse regression [36] was applied to flag any associations driven by the differential allele frequency of a control indication(s) rather than by IPF. In all studies, low frequency variants (MAF<1%) and variants with a poor imputation quality (r^2^<0.3) were removed from the analyses. A fixed-effect weighted meta-analysis combining the association results of the seven studies was performed using METAL [37] to establish the genetic variants associated with IPF. Variants were required to be present in at least two studies to be included in the analysis. The significance threshold was declared at p=4.50×10^−4^ after Bonferroni correction based on the number of independent SNPs, amino acid changes and classical HLA alleles in this region. The number of independent signals in the region was identified using LD-prune from PLINK v1.9 (r^2^=0.2) [38]. Conditional analyses were performed with GCTA-COJO [39] to identify independent sentinel variants. We used Meta-Analysis Model-Based Assessment of Replicability (MAMBA) to assess the consistency of association results across studies [40]. This tool calculates a posterior probability of replicability (PPR) that a given SNP has a non-zero replicable effect, indicating the likelihood of a specific SNP to replicate. Associations with a PPR >90% were considered consistent and likely to replicate. Variants were considered significant in our study if they not only satisfied the significance threshold of p=4.50×10^−4^ but also displayed significance at the nominal level in all seven studies or had a high probability or replication (PPR≥90%). The Manhattan and conditional analyses plots were obtained using HLA-TAPAS [41] and R v4.0.0 (www.r-project.org), respectively. Forest plots were performed with the *forestplot* R v4.1.3 package. Replication of the previous associations with HLA-DRB1*15:01 and HLA-DQB1*06:02 was sought in our meta-analysis results. For HLA-DQB1*06:02, we excluded the Colorado study from the analysis. Sensitivity analyses of all significant signals were conducted excluding the Colorado study, given that the case definition used included other fIIPs in addition to IPF.

## Results

**FIGURE 1:**
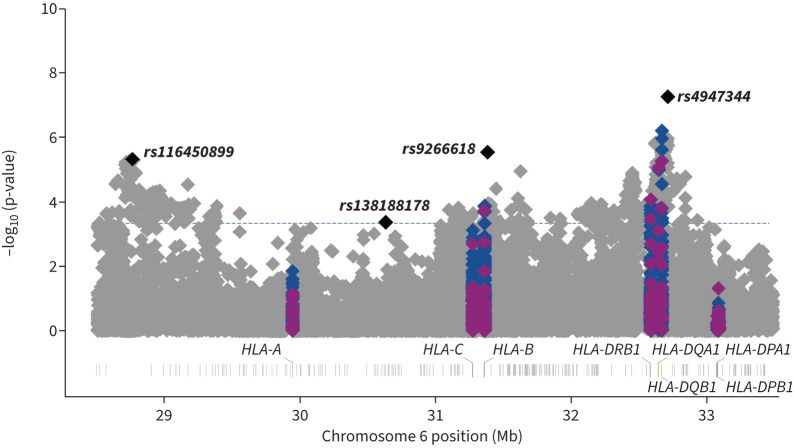
**Manhattan plot of meta-analysis results at chromosome 6 region**. Single nucleotide polymorphisms are shown in grey, amino acids in blue and classical human leukocyte antigen (HLA) alleles in purple. The four sentinel variants are highlighted in black. The y-axis shows the transformed p-values (–log_10_) while the x-axis represents chromosome positions in Mb (GRCh38/hg38). The horizontal line corresponds to the significance threshold of the study after Bonferroni correction (p=4.50×10^−4^).

**FIGURE 2:**
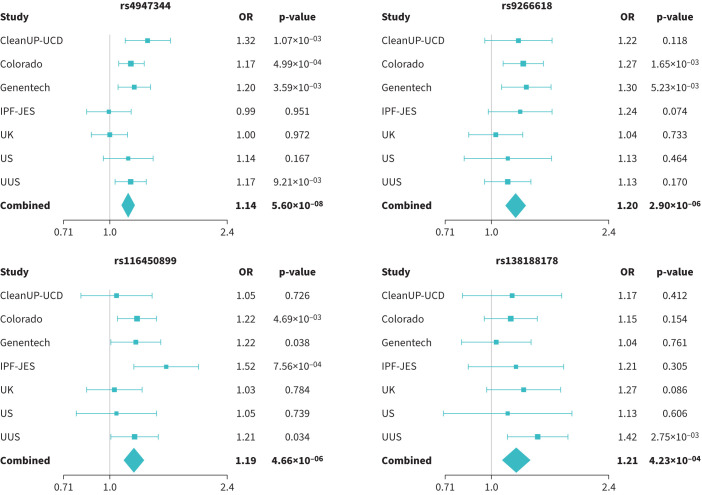
Forest plots of the association analysis results for the four sentinel variants.

A total of 32 618 unrelated individuals of European ancestry (5159 cases and 27 459 controls, supplementary table S1) and 44 713 common variants within the HLA region (43 544 SNPs, 174 classical alleles and 995 amino acid alleles) were included in the analyses. After meta-analysis, a total of 652 variants (comprising SNPs, classical HLA alleles and amino acid alleles) met the HLA-wide significance threshold of p=4.50×10^−4^ (figure 1). Subsequent conditional analysis revealed that these variants were located within four independent loci (table 1, supplementary table S3, figures 1 and 2).

**TABLE 1:** Association analysis results for sentinel variants

**Variant ID**	**Position (b38)**	**Nearest gene(s)**	**Non-effect allele**	**Effect allele**	**EAfreq**	**Direction** ^#^	**Study p≤0.05** ^#^	**OR (95%CI)**	**p-value**	**MAMBA** **(PPR %)**
**rs4947344**	chr6:2710069	*DQB1*/*HLA-DQA2*	C	T	0.286	+++−−++	YYYNNNY	1.14 (1.09–1.20)	5.60×10^−8^	0.85
**rs9266618**	chr6:31378389	*HLA-B*/*MICA*	A	C	0.093	+++++++	NYYNNNN	1.20 (1.11–1.29)	2.73×10^−6^	0.63
**rs116450899**	chr6:28764432	*ZBED9*/*TRIM27*	G	A	0.090	+++++++	NYYYNNN	1.19 (1.10–1.28)	4.65×10^−6^	1.31
**rs138188178**	chr6:30632465	*ATAT1*	G	T	0.043	+++++++	NNNNNNY	1.21 (1.09–1.34)	4.14×10^−4^	0.28

EAfreq: frequency of the effect allele across the entire sample; MAMBA: Meta-Analysis Model-Based Assessment of Replicability; PPR: posterior probability of replication; Y: yes; N: no. ^#^: order for effect Direction and Study significance is: CleanUP-UCD – Colorado – Genentech – IPF-JES – UK – US – UUS.

The top sentinel variant was the SNP rs4947344, an intergenic variant located between *HLA-DQB1* and *HLA-DQA2* that was associated with IPF risk in our study (OR 1.14, 95% CI 1.09–1.20, p=5.60×10^−8^). After conditional analyses, we found that this variant was in linkage disequilibrium with the alleles HLA-DQB1*06:02 and HLA-DRB1*15:01 (supplementary figure S1), previously associated with fIIP [5].

The three remaining sentinel variants identified in the meta-analysis results were SNPs rs9266618, rs116450899 and rs138188178 (table 1, figures 1 and 2). rs9266618 and rs116450899 are intergenic variants located between *HLA-B* and the MHC class I polypeptide-related sequence A (*MICA*), and between the zinc finger BED-type containing 9 (*ZBED9*) and the tripartite motif containing 27 (*TRIM27*) genes, respectively, while rs138188178 is intronic to the gene encoding ɑ-tubulin acetyltransferase 1 (*ATAT1*).

None of the variants met nominal significance (p<0.05) in all seven contributing studies and the replicability assessment with MAMBA revealed that none of these association signals was considered likely to replicate (PPR<90% in all cases) (table 1, figure 2).

After sensitivity analyses excluding the Colorado study, three signal associations reached the Bonferroni p-value threshold, although the likelihood of replication remained very small for all three (supplementary table S4). The association of HLA-DQB1*06:02 with IPF was not independently replicated in the six independent IPF datasets (p=0.043, PPR=0.09%) (supplementary table S4 and figure S2). While HLA-DRB1*15:01 reached the significance threshold in the meta-analysis (p=8.38×10^−5^), it was only nominally significant in the Colorado study, with a low probability of replication (PPR=0.35%) (supplementary table S5 and figure S3). Notably, the significance dropped after excluding the Colorado study from the analyses (p=0.135) (supplementary table S5).

## Discussion

In this study, we used genotype data and imputation of SNPs, amino acids and classical HLA alleles from seven independent case–control cohorts to test for association with IPF susceptibility. Although we identified four significant associations, including a signal correlated with a previously reported association for HLA-DQB1*06:02, the effects were inconsistent across the contributing studies and this heterogeneity was reflected in a poor PPR for all signals. The reason for this heterogeneity is unclear, but could be due to a broader definition of cases (including non-IPF fIIP) in the Colorado study [5] or use of a different methodology (whole-genome sequencing) in the Genentech study, among other possibilities. While control subjects in the Genentech study were selected from clinical trials of non-IPF diseases, including those strongly associated with variation in the HLA region (*e.g.* asthma), reverse regression on these four associations did not detect that the control cohort composition was driving the IPF effect estimates [18]. Recent studies also suggest that genetic variation within the HLA region may not be linked to IPF outcomes [15, 42].

We acknowledge several limitations. First, our study was restricted to individuals of European genetic ancestries. Thus, we cannot exclude the possibility that HLA variation might contribute to IPF susceptibility in individuals of other genetic ancestries. Next, the heterogeneity of signals across cohorts might suggest that HLA variation could contribute to forms of pulmonary fibrosis that do not meet the strict diagnostic criteria for IPF. Owing to technological limitations, we were unable to evaluate all types of classical HLA alleles, which could have concealed potential IPF associations. Additionally, the Bonferroni correction approach can be overly conservative, potentially masking weaker signals. Finally, the HLA is a complex region with known genetic and environmental interactions. Therefore, our approach of testing individual HLA variants with disease may be too simplistic to capture the complex interplay of these proteins with disease risk.

Our meta-analysis approach has the advantage of increasing the sample size for genetic studies of IPF. We restricted our analysis to studies of clinically defined IPF to avoid the issues of imprecise electronic healthcare record coding and resulting effect size attenuation that has been previously reported [43, 44]. By combining all available datasets, we were able to improve power to detect associations of large and modest effect sizes but at the expense of reserving samples for independent replication. To mitigate this, we used an approach that considers heterogeneity of association results across the meta-analysis to provide a PPR. This approach allowed us to provide a quantitative indicator of the combined strength of the associations across studies rather than relying on a single combined p-value that can be influenced by effects in individual studies.

Our results contribute to our understanding of IPF pathophysiology, strongly suggesting that individual genetic variants within the HLA are not associated with susceptibility to IPF in individuals with European ancestries, although they may have a role in other idiopathic interstitial pneumonias. Future studies investigating the role of infection in the aetiology of IPF should prioritise the assessment of interactions among immune system genes, with the aim of uncovering more promising pathways for therapeutic interventions in the care of these patients.

## Supplementary material

10.1183/23120541.00553-2023.Supp1 **Please note:** supplementary material is not edited by the Editorial Office, and is uploaded as it has been supplied by the author.
Supplementary material 00553-2023.SUPPLEMENT
