# Supplementary material for: Association study of human leukocyte antigen variants and idiopathic pulmonary fibrosis
Source: ERJ Open Res. 2024 Feb 19;10(1):00553-2023. doi: 10.1183/23120541.00553-2023 (PMC10875457; doi:10.1183/23120541.00553-2023)
Supplement: Supplementary file 1 [file 00553-2023.SUPPLEMENT.pdf]

Association study of human leukocyte antigen (HLA) variants and idiopathic pulmonary fibrosis

Supplemental material

Beatriz Guillen-Guio*, Megan L. Paynton*, Richard J. Allen, Daniel P.W. Chin, Lauren J. Donoghue, Amy Stockwell, Olivia C. Leavy, Tamara Hernandez-Beeftink, Carl Reynolds, Paul Cullinan, Fernando Martinez, CleanUP-IPF Investigators of the Pulmonary Trials Cooperative, Helen L. Booth, William A. Fahy, Ian P. Hall, Simon P. Hart, Mike R. Hill, Nik Hirani, Richard B. Hubbard, Robin J. McAnulty, Ann B. Millar, Vidya Navaratnam, Eunice Oballa, Helen Parfrey, Gauri Saini, Ian Sayers, Martin D. Tobin, Moira K. B. Whyte, Ayodeji Adegunsoye, Naftali Kaminski, Shwu-Fan Ma, Mary E. Strek, Yingze Zhang, Tasha E. Fingerlin, Maria Molina-Molina, Margaret Neighbors, X. Rebecca Sheng, Justin M. Oldham, Toby M. Maher, Philip L. Molyneaux, Carlos Flores, Imre Noth, David A. Schwartz, Brian L. Yaspan, R. Gisli Jenkins, Louise V. Wain[#], Edward J. Hollox[#].

*Equal contribution as first authors
[#]Equal contribution as senior authors

SUPPLEMENTARY METHODS 2
Study Cohorts 2
SUPPLEMENTARY TABLES 3
Table S1: Case definition and sample size of the study 3
Table S2. Statistical analysis sequence used in the study 3
Table S3: Effect allele frequencies in each study 4
Table S4: Sensitivity analyses of sentinel variants excluding the Colorado study 4
Table S5: Validation of previously reported IPF associations within the HLA region 4
SUPPLEMENTARY FIGURES 5
Figure S1. Conditional analysis on rs4947344 5
Figure S2. Forest plot of the validation analysis results for *HLA-DQB1*06:02* 5
Figure S3. Forest plot of the validation analysis results for *HLA-DRB1*15:01* 6
REFERENCES 7

SUPPLEMENTARY METHODS

Study Cohorts

We analysed genomic data from seven previously described independent case-control studies of idiopathic pulmonary fibrosis (IPF). In all seven studies, IPF cases were diagnosed according to the American Thoracic Society and European Respiratory Society guidelines (Table S1) (1-5).

The Study of Clinical Efficacy of Antimicrobial Therapy Strategy Using Pragmatic Design in Idiopathic Pulmonary Fibrosis - University of California Davis (CleanUP-UCD) comprised a total of 469 IPF cases from a randomized clinical trial conducted across 35 US locations and from the University of California Davis (6,7), as well as 2,455 population controls selected from UK Biobank. Both cases and controls were genotyped using the Affymetrix UK Biobank array.

The Colorado study (8) included 1,515 patients with fibrotic idiopathic interstitial pneumonias (fIIP) from US cohorts (National Jewish Health IIP population, InterMune IPF trials, UCSF, Vanderbilt University IIP population, and the National Heart Lung and Blood Institute Lung Tissue Research Consortium) and 2,455 population controls selected so they were genetically similar to the cases. All individuals were genotyped using the Illumina Human 660W Quad BeadChip array.

The Genentech study (9,10) consisted of 813 cases from three IPF clinical trials (ASCEND, CAPACITY, and RIFF) and 3,949 controls from non-IPF clinical trials for age-related macular degeneration, diabetic macular oedema, multiple sclerosis, asthma, and inflammatory bowel disease. Genotypes were obtained from whole-genome sequencing using HiSeq X Ten platform (Illumina Inc.) to an average read depth of 30X.

The Idiopathic Pulmonary Fibrosis Job Exposures Study (IPF-JES) (11) included 416 men from England, Scotland or Wales diagnosed with IPF and 2,465 matching controls (all men) selected from both the IPF-JES study (individuals with an outpatient clinic appointment during the study period) and UK Biobank. Genotyping was performed with the Affymetrix UK Biobank array.

The UK study (12) comprised a total of 612 IPF cases from 9 different centres across the UK and 3,366 matching controls selected from UK Biobank. Cases were genotyped with the Affymetrix UK BILEVE array and controls with the UK Biobank array.

The US study (13) included 541 IPF cases from the University of Chicago, University of Pittsburgh and COMET study, and 542 population controls from the database of genotypes and phenotypes (dbGaP) and the University of Pittsburgh, all genotyped with the Affymetrix Genome-Wide Human SNP 6.0 array.

The UUS study [United States, United Kingdom, and Spain] (14) consisted of 793 IPF cases from 7 study cohorts (ACE, PANTHER, UCD, Chicago, UCSF, PROFILE, and Spain) and 9,999 population controls selected from UK Biobank matching ancestry, sex and smoking distribution. Cases were genotyped with the Affymetrix UK Biobank and Spain Biobank arrays, and controls with the UK Biobank array.

All studies were performed in accordance with The Code of Ethics of the World Medical Association (Declaration of Helsinki) and approved by the appropriate institutional review or Research Ethics Committee.

SUPPLEMENTARY TABLES

Table S1: Case definition and sample size of the study

	Case definition*	N Cases	N Controls	N Total
CleanUP-UCD	IPF (4,5)	469	2,455	2,924
Colorado	fIIP (including IPF) (2)	1,515	4,683	6,198
Genentech	IPF (3)	813	3,949	4,762
IPF-JES	IPF (3)	416	2,465	2,881
UK	IPF (2-4)	612	3,366	3,978
US	IPF (1,3)	541	542	1,083
UUS	IPF (2,3,5)	793	9,999	10,792
**Total**		**5,159**	**27,459**	**32,618**

fIIP: fibrotic idiopathic interstitial pneumonia, IPF: idiopathic pulmonary fibrosis, N: number of individuals after quality controls. *All IPF cases were diagnosed according to the American Thoracic Society and European Respiratory Society guidelines (1-5).

Table S2. Statistical analysis sequence used in the study

		Colorado, CleanUP-UCD, IPF-JES, UK, US, and UUS studies	Genentech
1. Variant imputation	Single nucleotide polymorphisms	TOPMed (15) and T1DGC (16) reference panels	Whole-genome sequencing data
	Classical HLA alleles and amino acids	T1DGC reference panel	HLA-HD algorithm (17)
2. Association analyses	Method	Logistic regression in each study separately	Logistic regression
	Covariates	10 PC	Sex, age and 5 PCs
3. Meta-analysis	Method	Fixed effects weighted meta-analysis	
	Variant filtering	- Low frequency (MAF < 1%)<br>- Poor imputation quality (r2 < 0.3)<br>- Present in only one of the seven studies	
4. Significance criteria		HLA-wide significance threshold of p=4.50×10^{-4}<br>AND<br>Nominal significance in all studies OR Posterior Probability of Replication (PPR) ≥90% according to MAMBA (18)	

MAF, minor allele frequency; MAMBA, Meta-Analysis Model-Based Assessment of Replicability; PC, Principal components; T1DGC, type one diabetes genomics consortium.

Table S3: Effect allele frequencies in each study

Variant ID	Position (b38)	NEA/EA	EAfreq Meta	EAfreq / Imp.Q						
				CleanUP-UCD	Colorado	Genentech	IPF-JES	UK	US	UUS
rs4947344	chr6:32710069	C/T	0.286	0.293 / 0.991	0.290 / 0.987	0.281 / NA	0.281 / 0.991	0.283 / 0.997	0.290 / 0.984	0.280 / 0.992
rs9266618	chr6:31378389	A/C	0.093	0.100 / 0.979	0.083 / 0.959	0.083 / NA	0.097 / 0.979	0.103 / 0.983	0.084 / 0.898	0.102 / 0.972
rs116450899	chr6:28764432	G/A	0.089	0.092 / 0.999	0.093 / 0.998	0.083 / NA	0.088 / 0.999	0.091 / 0.999	0.090 / 0.996	0.087 / 0.999
rs138188178	chr6:30632465	G/T	0.043	0.044 / 0.989	0.044 / 0.99	0.046 / NA	0.04 / 0.985	0.043 / 0.995	0.036 / 0.894	0.043 / 0.987

NEA, non-effect allele; EA, effect allele; EAfreq, frequency of the effect allele. Imp.Q, Imputation quality.

Table S4: Sensitivity analyses of sentinel variants excluding the Colorado study

Variant ID	Position (b38)	NEA/EA	Results excluding Colorado		
			OR [95%CI]	P	PPR
rs4947344	chr6:32710069	C/T	1.13 [1.07,1.20]	2.43×10^{-5}	1.24%
rs9266618	chr6:31378389	A/C	1.17 [1.07,1.28]	3.52×10^{-4}	0.63%
rs116450899	chr6:28764432	G/A	1.18 [1.08,1.29]	2.83×10^{-4}	1.88%
rs138188178	chr6:30632465	G/T	1.23 [1.08,1.39]	1.10×10^{-3}	0.88%

NEA, non-effect allele; EA, effect allele. Associations reaching the Bonferroni threshold (p=4.50×10^{-4}) are shaded in grey. PPR, posterior probability of replication (obtained with MAMBA, Meta-Analysis Model-Based Assessment of Replicability).

Table S5: Validation of previously reported IPF associations within the HLA region

Variant ID	Position (b38)	Meta-analysis			Excluding Colorado		
		OR [95%CI]	P	PPR	OR [95%CI]	P	PPR
*HLA-DQB1**06:02	chr6:32663284	1.16 [1.09, 1.23]	5.66×10^{-6}	0.64%	1.08 [1.00, 1.16]	0.043	0.09%
*HLA-DRB1**15:01	chr6:32584287	1.13 [0.06, 0.19]	8.38×10^{-5}	0.35%	1.06 [-0.02, 0.132]	0.135	0.08%

PPR, posterior probability of replication (obtained with MAMBA, Meta-Analysis Model-Based Assessment of Replicability).

SUPPLEMENTARY FIGURES

Figure S1. Conditional analysis on rs4947344

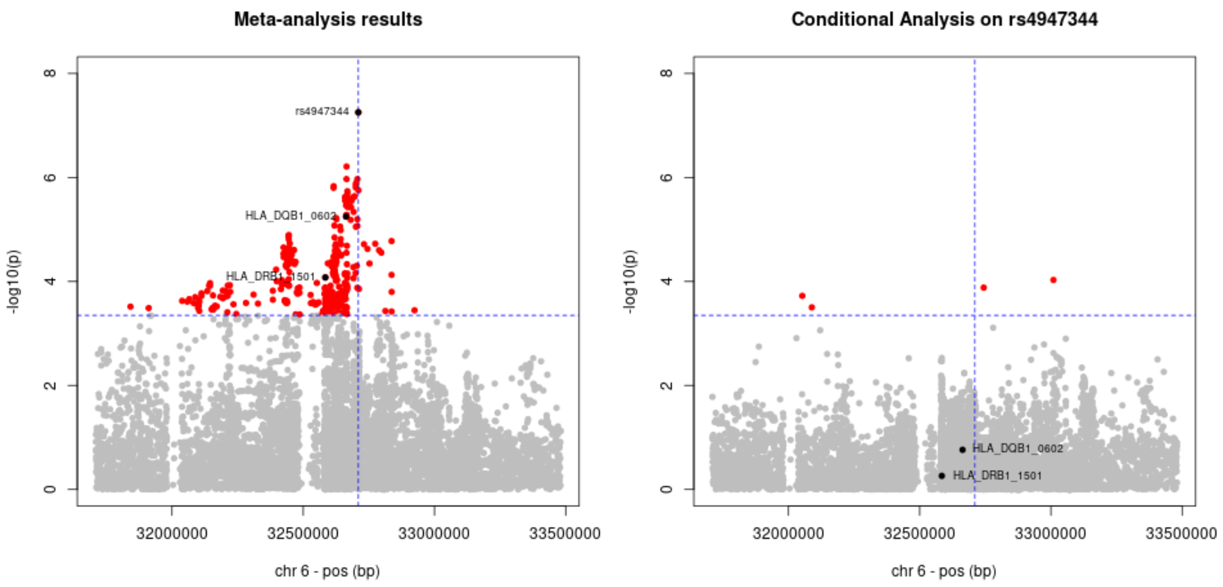

Figure S2. Forest plot of the validation analysis results for *HLA-DQB1*06:02*

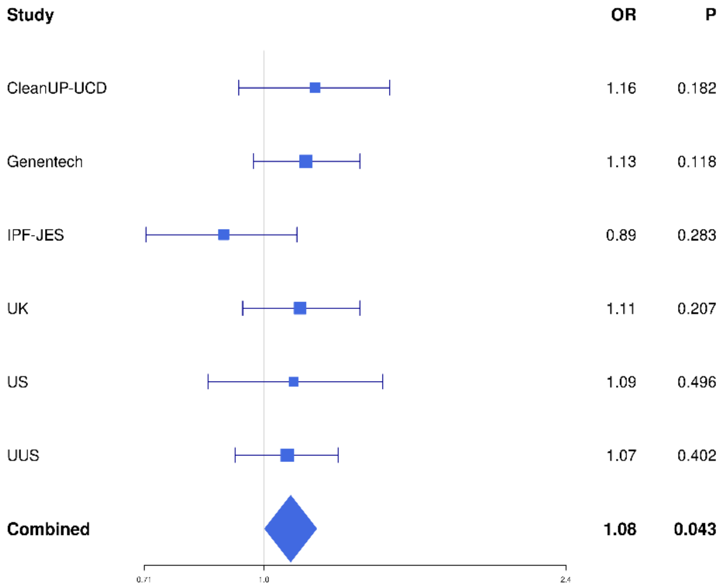

Figure S3. Forest plot of the validation analysis results for *HLA-DRB1*15:01*

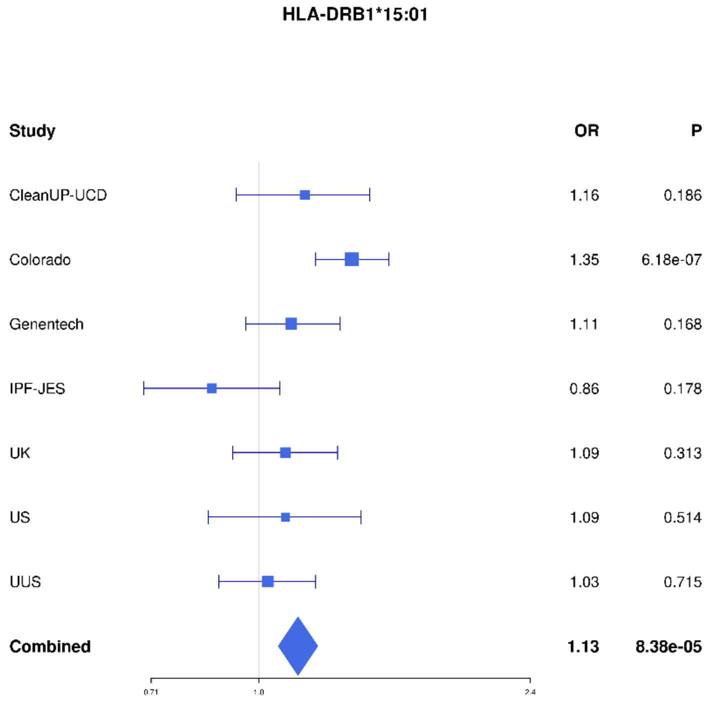

REFERENCES

1. American Thoracic S, European Respiratory S. American Thoracic Society. Idiopathic pulmonary fibrosis: diagnosis and treatment. International consensus statement. American Thoracic Society (ATS), and the European Respiratory Society (ERS). *Am J Respir Crit Care Med* 2000;**161**(2 Pt 1):646-64.
2. American Thoracic S, European Respiratory S. American Thoracic Society/European Respiratory Society International Multidisciplinary Consensus Classification of the Idiopathic Interstitial Pneumonias. This joint statement of the American Thoracic Society (ATS), and the European Respiratory Society (ERS) was adopted by the ATS board of directors, June 2001 and by the ERS Executive Committee, June 2001. *Am J Respir Crit Care Med* 2002;**165**(2):277-304.
3. Raghu G, Collard HR, Egan JJ, et al. An official ATS/ERS/JRS/ALAT statement: idiopathic pulmonary fibrosis: evidence-based guidelines for diagnosis and management. *Am J Respir Crit Care Med* 2011;**183**(6):788-824.
4. Raghu G, Rochwerg B, Zhang Y, et al. An Official ATS/ERS/JRS/ALAT Clinical Practice Guideline: Treatment of Idiopathic Pulmonary Fibrosis. An Update of the 2011 Clinical Practice Guideline. *Am J Respir Crit Care Med* 2015;**192**(2):e3-19.
5. Raghu G, Remy-Jardin M, Myers JL, et al. Diagnosis of Idiopathic Pulmonary Fibrosis. An Official ATS/ERS/JRS/ALAT Clinical Practice Guideline. *Am J Respir Crit Care Med* 2018;**198**(5):e44-e68.
6. Martinez FJ, Yow E, Flaherty KR, et al. Effect of Antimicrobial Therapy on Respiratory Hospitalization or Death in Adults With Idiopathic Pulmonary Fibrosis: The CleanUP-IPF Randomized Clinical Trial. *JAMA* 2021;**325**(18):1841-51.
7. Allen RJ, Oldham JM, Jenkins DA, et al. Longitudinal lung function and gas transfer in individuals with idiopathic pulmonary fibrosis: a genome-wide association study. *Lancet Respir Med* 2023;**11**(1):65-73.
8. Fingerlin TE, Murphy E, Zhang W, et al. Genome-wide association study identifies multiple susceptibility loci for pulmonary fibrosis. *Nat Genet* 2013;**45**(6):613-20.
9. Dressen A, Abbas AR, Cabanski C, et al. Analysis of protein-altering variants in telomerase genes and their association with MUC5B common variant status in patients with idiopathic pulmonary fibrosis: a candidate gene sequencing study. *Lancet Respir Med* 2018;**6**(8):603-14.
10. Donoghue LJ, Stockwell AD, Neighbors M, et al. Identification of a Genetic Susceptibility Locus for Idiopathic Pulmonary Fibrosis in the 16p Subtelomere Using Whole-Genome Sequencing. *Am J Respir Crit Care Med* 2023;**207**(7):941-44.
11. Reynolds CJ, Sisodia R, Barber C, et al. What role for asbestos in idiopathic pulmonary fibrosis? Findings from the IPF job exposures case-control study. *Occup Environ Med* 2023;**80**(2):97-103.
12. Allen RJ, Porte J, Braybrooke R, et al. Genetic variants associated with susceptibility to idiopathic pulmonary fibrosis in people of European ancestry: a genome-wide association study. *Lancet Respir Med* 2017;**5**(11):869-80.
13. Noth I, Zhang Y, Ma SF, et al. Genetic variants associated with idiopathic pulmonary fibrosis susceptibility and mortality: a genome-wide association study. *Lancet Respir Med* 2013;**1**(4):309-17.
14. Allen RJ, Guillen-Guio B, Oldham JM, et al. Genome-Wide Association Study of Susceptibility to Idiopathic Pulmonary Fibrosis. *Am J Respir Crit Care Med* 2020;**201**(5):564-74.
15. Taliun D, Harris DN, Kessler MD, et al. Sequencing of 53,831 diverse genomes from the NHLBI TOPMed Program. *Nature* 2021;**590**(7845):290-99.
16. Jia X, Han B, Onengut-Gumuscu S, et al. Imputing amino acid polymorphisms in human leukocyte antigens. *PLoS One* 2013;**8**(6):e64683.
17. Kawaguchi S, Higasa K, Shimizu M, et al. HLA-HD: An accurate HLA typing algorithm for next-generation sequencing data. *Hum Mutat* 2017;**38**(7):788-97.

18. McGuire D, Jiang Y, Liu M, et al. Model-based assessment of replicability for genome-wide association meta-analysis. *Nat Commun* 2021;**12**(1):1964.
